# Pupil responses to melanopsin-isolating stimuli as a potential diagnostic biomarker for glaucoma

**DOI:** 10.1371/journal.pone.0324373

**Published:** 2025-05-23

**Authors:** Jonathan Denniss, Riccardo Cheloni, Joel T. Martin, Manuel Spitschan

**Affiliations:** 1 School of Optometry & Vision Science, University of Bradford, Bradford, United Kingdom; 2 Moorfields Eye Hospital NHS Foundation Trust, London, United Kingdom,; 3 School of Psychology, Philosophy and Language Sciences, University of Edinburgh, Edinburgh, United Kingdom; 4 Max Planck Research Group Translational Sensory & Circadian Neuroscience, Max Planck Institute for Biological Cybernetics, Tübingen, Germany; 5 TUM School of Medicine and Health, Department Health and Sports Sciences, Chronobiology & Health, Technical University of Munich, Munich, Germany; 6 TUM Institute for Advanced Study (TUM-IAS), Technical University of Munich, Garching, Germany; Transilvania University of Brasov: Universitatea Transilvania din Brasov, ROMANIA

## Abstract

**Purpose:**

To test whether differences in pupil responses to melanopsin-isolating spectral stimuli in glaucoma may be useful as a diagnostic biomarker.

**Methods:**

Spectral stimuli were presented to 20 glaucoma and 15 age-similar healthy control participants. Stimuli were pairs of silent-substitution spectra designed to provide (1) equal stimulation to cone photoreceptors but maximum (~325%) contrast to melanopsin or (2) equal stimulation to melanopsin but ~325% contrast to cones. Narrowband long-wavelength/red (657 nm) and short-wavelength/blue (471 nm) pulses were also presented from a dark background to 16 glaucoma and 12 control participants. Pulses lasted 3 seconds and pupil size was measured for 15 seconds. Pupil response metrics were compared by t-test and relationships with visual field and OCT summary indices were assessed by Spearman’s rank correlation. Diagnostic accuracy was measured by area under the receiver operating characteristic curve (AUC).

**Results:**

Pupil constriction was more persistent after pulse offset for the melanopsin-directed stimulus (2% mean paired difference 6s post-pulse offset, p < 0.001). All pupil parameters were similar between groups (p = 0.04–0.90) for all stimuli. Correlations between pupil response parameters and visual field summary indices and circumpapillary retinal nerve fibre layer thickness were weak (rho 0.02–0.57, all p > 0.05). Diagnostic accuracy for all pupil parameters was poor, with AUC 95% confidence intervals overlapping 0.5 for all but time to maximal constriction for the cone-directed stimulus.

**Conclusions:**

Pupil responses to melanopsin-isolating spectra were similar between glaucoma and control participants. Pupillary responses to melanopsin-isolating silent substitution spectra are unlikely to be useful as a diagnostic biomarker for glaucoma.

## Introduction

Glaucoma causes death of retinal ganglion cells (RGCs), leading to irreversible loss of vision. The intrinsically photosensitive RGCs (ipRGCs) are a sparsely-distributed subtype of RGCs in the human retina that express the photopigment melanopsin. Whilst some ipRGCs receive synaptic input from rods and cones, the melanopsin photopigment additionally makes ipRGCs independently sensitive to short-wavelength light [1,2]. The exact function of ipRGCs in humans remains a topic of investigation, but they are known to contribute primarily to non-image forming vision, including the pupillary light reflex and the synchronisation of the circadian clock to the environmental light-dark cycle [1,2].

Histological evidence suggests that ipRGCs are lost in glaucoma, similar to other RGC subtypes [3]. Loss of ipRGCs is unlikely to be detected by clinical visual field tests due to their minimal contribution to image-forming vision, nor by optical coherence tomography due to their low number relative to other RGC classes. Loss or dysfunction of ipRGCs may, however, manifest as alterations to the post-illumination pupil response (PIPR), which is thought to reflect largely melanopsin signals. Several studies have shown reductions in the group-mean PIPR in experimental participants with glaucoma as compared to age-similar healthy control participants [4–9]. One study has further attempted to localise the stimulus to retinal quadrants and measure diagnostic accuracy, showing promising results for detection of glaucoma [7]. These previous studies have used narrowband red and blue lights to stimulate pupil reactions, finding a reduced PIPR in glaucoma compared to control groups. A limitation of these previous studies is that narrowband light of any wavelength will always activate all photoreceptors because photoreceptor spectral sensitivities are highly overlapping [10]. This imperfect isolation means that the measured pupil response in previous studies is not specific to the melanopsin-driven component of ipRGC responses, and their loss of function in glaucoma may be partly masked as a result.

Previous studies of the healthy visual system have used silent substitution methods to produce stimuli that more accurately isolate the response of melanopsin, and, therefore, the ipRGCs from those of cone photopigments [10–13]. In this study, we use the method of silent substitution to measure pupil responses to melanopsin-directed stimuli in human participants with glaucoma and age-similar healthy controls. We hypothesised that by isolating melanopsin in this way, we would be able to measure differences in pupil response between participants with glaucoma and healthy controls, and aimed to test whether this technique has potential as a clinical diagnostic test.

## Methods

We measured pupil responses to melanopsin-directed and L-M-S-cone (LMS) photoreceptor-directed (henceforth termed LMS-directed) stimuli in participants with glaucoma and age-similar healthy control participants. Melanopsin-directed stimuli consisted of stimulus pairs with matched contrast on LMS-cone photopigments (<6% absolute contrast) but maximal contrast (~325%) on melanopsin. LMS-directed stimuli were presented as a control, and consisted of stimulus pairs matched for their contrast on melanopsin (<6% absolute contrast) whilst matching their contrast on LMS to that of the melanopsin-directed stimuli on melanopsin (~325%). We additionally measured pupil responses to narrowband red (657 nm) and blue (471 nm) stimuli, similar to previous studies, in a subset of our participants. See *Apparatus & Stimuli* later for further details of the stimuli used.

## Participants

Participants with glaucoma and age-similar healthy control participants were recruited from a database of those who had previously attended or registered interest for studies conducted at the University of Bradford. Participants with glaucoma had open-angle glaucoma confirmed by either written diagnosis from a consultant ophthalmologist or self-report with evidence of current glaucoma treatment. All participants with glaucoma had a circumpapillary retinal nerve fibre layer (cpRNFL) defect defined as at least one sector with p < 1% on Spectralis (Heidelberg Engineering GmbH, Germany) optical coherence tomography (OCT), and a visual defect defined as Mean Deviation p < 5% and/or 3 contiguous non-edge points with Pattern Deviation p < 5% and/or Glaucoma Hemifield Test outside normal limits on a SITA standard 24–2 visual field test (Humphrey Field Analyzer 3, Carl Zeiss Meditec, Jena, Germany). Healthy control participants had neither cpRNFL defect nor visual field defect, measured in the same way as for the glaucoma group, and had intraocular pressure below 21 mmHg in both eyes. Intraocular pressure was initially measured by non-contact tonometry (Pulsair, Keeler, Windsor, UK, average of 4 repeats) then repeated with Goldmann applanation tonometry if either eye was > 21 mmHg or the between-eye difference exceeded 4 mmHg on non-contact tonometry.

Common inclusion criteria for both groups were refractive error between -6.00DS and +6.00DS and less than -3.00DC, corrected visual acuity better than 6/9.5, normal colour vision as measured with the City University test [14], either history of uncomplicated cataract surgery with clear intraocular lens or no significant lens opacity defined as nuclear opacity <=3, cortical cataract <=2, posterior subcapsular cataract <=2 on the LOCSIII grading scale [15], clinically normal direct and consensual pupil reactions in both eyes, slit lamp examination showing intact iris with no tears and round pupil free from any visible pathological process. All participants had no sign or history of eye or systemic disease that could affect vision or pupil reactions other than glaucoma for those in the glaucoma group. Participants were not excluded for diagnosis of type II diabetes if there were no ocular signs. No participant was currently using any medication known to affect pupil responses. One eye was tested per participant, selected at random if both eyes were eligible.

All participants provided written informed consent to take part in accordance with the Declaration of Helsinki. The study received ethical approval from the National Health Service Research Ethics Service. Participants were recruited from 8^th^ November 2021–3^rd^ February 2022.

### Apparatus & Stimuli

Light stimuli were prepared and administered using the system of hardware and software described by Martin et al. [16] which briefly comprises a spectrally tuneable 10-primary light source (Spectra Tune Lab, LEDMOTIVE Technologies, LLC, Barcelona, Spain), a 45-cm diameter full-field integrating sphere, a head-mounted eye tracker (Pupil Core, Pupil Labs GmbH, Berlin, Germany), and a suite of open-source Python software [17] for generating stimuli and controlling the devices. The spectral response of the system was measured at the corneal plane across the 12-bit input range for each primary using a spectroradiometer (Spectraval 1501, JETI Technische Instrumente, GmbH, Jena, Germany). These data, shown in S1 Fig, were subsequently used to generate LMS- and melanopsin-directed stimuli in a constrained numerical optimisation procedure that aimed to maximise contrast on the targeted photoreceptor(s) whilst minimising contrast on the remaining photoreceptor(s). This optimisation was performed in Python using SciPy’s ‘basinhopping’ global search algorithm [18]. We first determined settings for a stimulus pair to maximise contrast on melanopsin whilst maintaining equal stimulation on LMS cones. Next, we determined settings for a stimulus pair that maximised contrast on LMS cones whilst maintaining equal stimulation on melanopsin, with the additional constraint of matching overall LMS contrast to the melanopsin contrast in the melanopsin-directed stimulus pair. Further details of the silent substitution method are given by Spitschan & Woelders [10].

**Fig 1:**
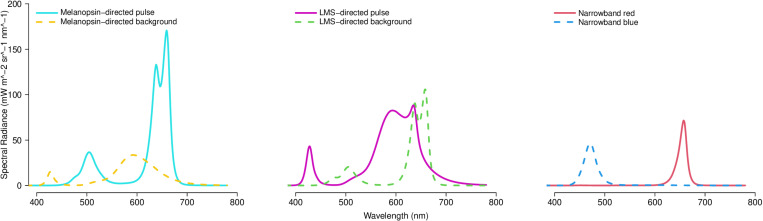
Spectral radiance of each stimulus pulse and background.

Because there was a small amount of variation in the spectral output of our system, we took 5 spectral radiance measurements of each stimulus both before and after each data collection session. The means of all of these measurements for each stimulus are shown in Fig 1 and are provided in the Supplementary Materials. We also used these measurements to calculate the expected radiance Weber contrast on melanopsin, L-cone-opsin, M-cone-opsin and S-cone-opsin for both the melanopsin-directed and LMS-directed stimulus pairs. Weber contrasts (in %) for each opsin were calculated as 100*(pulse radiance – background radiance)/ background radiance. Radiances for each opsin were calculated using the open-source software platform *luox* [19,20] incorporating the CIE S026 spectral sensitivity functions for 10° field. [21] Table 1 shows the achieved contrast on each opsin and the overall luminance of the pulse and background for each stimulus pair.

**Table 1:** Median (interquartile range) radiance Weber contrast achieved on each opsin, and median (interquartile range) overall luminance of the melanopsin- and LMS-directed stimulus pairs.

	Melanopsin-directed stimulus	LMS-directed stimulus
**Radiance Weber contrast (%)**		
Melanopsin	325.0 (323.5 to 327.5)	-5.0 (-5.3 to -4.9)
L-cone-opsin	0.1 (-0.3 to 1.0)	325.7 (323.2 to 327.0)
M-cone-opsin	0.0 (-0.3 to 0.4)	338.1 (336.5 to 338.9)
S-cone-opsin	-5.8 (-6.4 to -5.6)	277.1 (276.9 to 277.8)
**Overall luminance (cd/m**^**2**^)		
Pulse	1146 (1140–1155)	3129 (3118–3138)
Background	1227 (1225–1228)	678.7 (674.8 to 684.0)

Stimuli were presented monocularly to the tested eye whilst pupil size was simultaneously recorded at 120 Hz by an infrared eye tracking pupillometer (Pupil Core, Pupil Labs GmbH, Berlin, Germany). The fellow eye was carefully patched ensuring that no light could enter. At the centre of the integrating sphere there was a 7 cm diameter circular aperture through which an 11.4° diameter fixation cross was displayed. This aimed to reduce the effects of light absorption by macular pigmentation, which varies across the visual field [22], as well as facilitating steady fixation for better pupil tracking.

Narrowband red (peak wavelength 657 nm, full width at half maximum [FWHM] 17 nm) and blue (peak wavelength 471 nm, FWHM 27 nm) stimuli were generated by matching primaries 9 and 3 respectively S1 Fig for unweighted total radiance between 380 and 780 nm, whilst all remaining primaries were set to 0. The red stimulus had luminance 151 cd/m^2^ and the blue stimulus had luminance 117 cd/m^2^ (2.18 and 2.07 log_10_ cd/m^2^ respectively). Park et al. previously showed that similar stimuli with luminance 1 log_10_ cd/m^2^ or greater are sufficient to drive the human PIPR [23].

### Procedure

The protocol was similar to that described previously [13]. For each participant, stimuli were presented in blocks of 30 trials, broken down into three runs of 10 trials with short breaks in-between each run. For the red/blue stimuli 15 trials of each stimulus were presented in random order across the block of 30 trials. Red/blue stimuli were always presented first in the session, followed by LMS-directed stimuli and then melanopsin-directed stimuli last. All blocks started with a 4.5 minute background adaptation period in which the background spectra was presented for the melanopsin- and LMS-directed stimuli and no light was presented for the red/blue stimuli (i.e., dark adaptation). Individual trials started with a 400–600 ms dark flash which enabled temporal synchronisation between the light source and pupil tracker. Background illumination as above was then restored for a pre-pulse interval of random duration between 10 and 12s prior to the 3s stimulus pulse [13], followed by a return to background illumination for a 15s post-pulse period. After a run of 10 trials had been completed, participants took a break of approximately 1 minute before the next run was initiated with a 30s background/dark re-adaptation period. A break of up to 5 minutes was taken between blocks. We did not include longer dark adaptation because initial pilot testing showed no difference in pupil responses with or without 30 minutes of dark adaptation and a requirement for long periods of dark adaptation would limit clinical applicability of the protocol. Total testing time was approximately 90 minutes including breaks. Participants were asked to avoid blinking for the duration of each trial.

### Data Analysis

As described previously [16], raw pupillometer data were initially converted to a 3-D model of pupil diameter by the pupillometer’s in-built software. Fitted pupil diameters were then processed in Python to remove blinks. Data were masked if the first derivative of the pupil size exceeded + /-3 standard deviations or if the corresponding confidence value provided by the pupillometer software was below 0.95. Masked data were then reconstructed by linear interpolation, before all data were smoothed with a third-order Butterworth filter with 4 Hz cut-off [16].

Pupil diameters recorded for each light stimulus presentation were normalised by conversion to percentage of baseline. Baseline was defined as the mean pupil diameter within 1s prior to stimulus onset.

**Fig 2:**
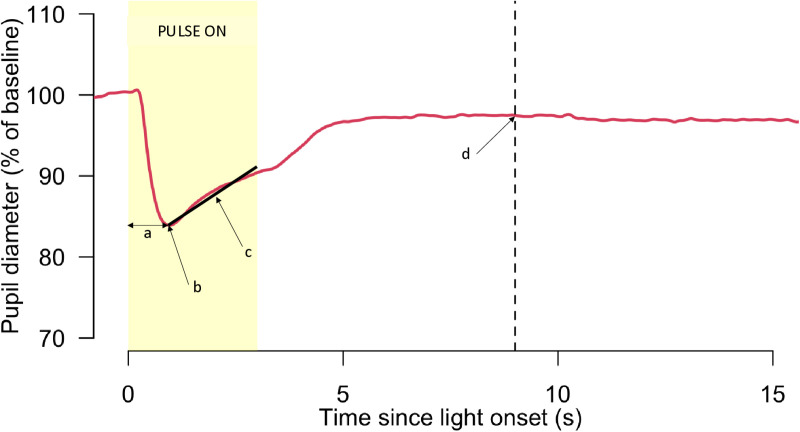
Example of the general form of the pupil response data to melanopsin- and cone-targeted stimuli. Labels a-d indicate the parameters analysed, as described in the text. The yellow box indicates the time during which the stimulus pulse was on (3s duration).

Pupil responses to both melanopsin- and LMS-directed stimuli take the general form exemplified in Fig 2. We analysed four parameters of the pupil response, as labelled a-d in Fig 2: (a) time taken to reach maximum pupil constriction from pulse onset, (b) pupil diameter at maximum constriction within pulse on period, (c) mean rate of pupil redilation from maximum constriction whilst pulse on (slope of linear fit to pupil diameter within this period) and (d) pupil diameter 6 s after pulse offset.

**Fig 3:**
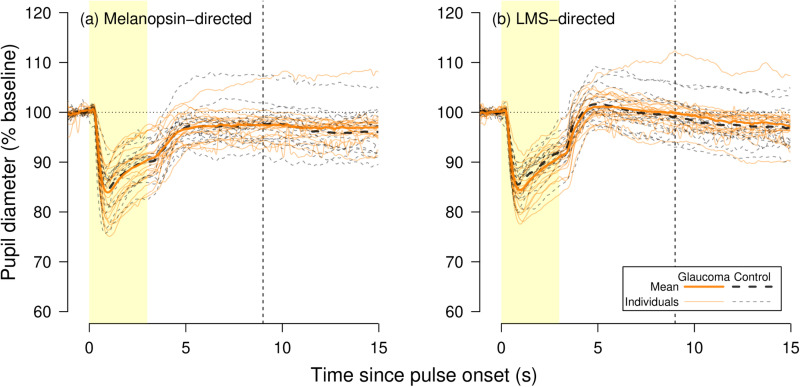
Pupil responses to (a) melanopsin-directed and **(b)** LMS-directed stimuli. Solid lines represent glaucoma participants, dashed lines represent control participants. Bold lines are group means, while fainter lines are individual participants’ mean responses. The yellow box indicates the period during which the stimulus pulse is on. Vertical dashed lines indicate 6s post pulse offset. Horizontal dotted lines indicate baseline pupil diameter.

For the red/blue stimuli, PIPR was calculated as the difference in pupil diameter 6s post stimulus offset between red and blue light stimuli (red-blue). As a secondary analysis, we also found the timepoint of maximal difference in PIPR between glaucoma and control participants. The PIPRs at both timepoints were compared between glaucoma and control participants by Welch’s t-test.

Mean pupil response parameters for all stimuli were compared between groups by Welch’s t-test. Mean pupil response parameters were compared between the melanopsin-directed and LMS-directed stimuli by paired t-test. Spearman’s correlation was used to assess relationships between each pupil response parameter for all stimuli and visual field Mean Deviation, Pattern Standard Deviation and mean circumpapillary retinal nerve fibre layer thickness from OCT in the glaucoma group. Finally, receiver operating characteristic (ROC) analysis was used to assess diagnostic performance for all parameters in separating glaucoma from controls. Statistical significance was assumed at p < 0.05, except where multiple comparisons are made, in which case family-wise Bonferroni correction was applied (e.g., for 4 comparisons, p < 0.0125 was considered statistically significant). Data were analysed in R (version 4.2.1), using the pROC package for ROC analysis [24].

## Results

Data were obtained from 20 glaucoma participants and 15 control participants for the melanopsin-directed and LMS-directed stimuli. 16 of the glaucoma participants and 12 of the control participants also contributed data for the narrowband red/blue stimuli. 8 of the glaucoma participants and 2 of the control participants had artificial intraocular lenses following cataract surgery. Table 2 summarises the demographic and clinical screening information for all participants.

**Table 2:** Demographic and clinical screening information for included participants. Continuous data are presented as mean (standard deviation). VF = visual field, cpRNFLT = circumpapillary retinal nerve fibre layer thickness.

	Glaucoma (n = 20)	Control (n = 15)
Age (years)	70.9 (6.0)	70.9 (4.6)
Male/Female	11/9	8/7
Best corrected visual acuity (logMAR)	-0.02 (0.06)	-0.03 (0.09)
VF Mean Deviation (dB)	-5.72 (2.67)	0.52 (0.62)
VF Pattern Standard Deviation (dB)	7.67 (2.88)	1.43 (0.21)
Mean cpRNFLT (microns)	62.8 (8.6)	96.2 (7.6)

Boxplots of pupil diameters for each group/stimulus during the baseline period of 1s prior to stimulus onset are provided in S2 Fig. Fitted and processed pupil measurements for each participant/stimulus are provided in the Supplementary Materials. Fig 3 shows the pupil responses to melanopsin- and LMS-directed stimuli for all participants.

As shown in Fig 3 the differences between glaucoma and control participants were small for both stimuli and none reached statistical significance at p < 0.0125 after correcting for family-wise multiple [4] comparisons. Between-group comparisons in all parameters are shown Table 3.

**Table 3:** *Between-group comparisons in measured parameters (a-d in* Fig 1*) for melanopsin- and LMS-directed stimuli. Values are mean (95% confidence interval). P-values are from Welch’s t-tests.*

	Glaucoma	Control	p
Melanopsin-directed stimulus:			
(a)Time to max. constriction (ms)	965 (904-1026)	1003 (763-1243)	0.75
(b)Diameter at max. constriction (%)	83.6 (81.3-85.9)	84.2 (81.7-86.6)	0.73
(c)Redilation rate (%s^-1^)	3.33 (2.70-3.95)	2.93 (2.12-3.74)	0.42
(d)Diameter 6s post-offset (%)	97.5 (96.1-98.8)	97.6 (95.5-99.8)	0.90
LMS-directed stimulus:			
(a)Time to max. constriction (ms)	990 (930-1049)	898 (834-963)	0.04
(b)Diameter at max. constriction (%)	84.1 (82.3-85.9)	85.3 (83.6-87.0)	0.31
(c)Redilation rate (%s^-1^)	3.43 (2.85-4.01)	3.17 (2.40-3.94)	0.57
(d)Diameter 6s post-offset (%)	99.8 (98.1-101.5)	99.2 (97.1-101.2)	0.63

There were differences in the form of the response between the melanopsin- and LMS-directed stimuli with more persistent pupil constriction after pulse offset for the melanopsin-directed stimulus (mean paired difference in pupil diameter at 6s post pulse offset 2%, p < 0.001). No other parameters showed statistically significant differences between stimuli (time to maximum constriction within pulse on period mean paired difference 31ms, p = 0.54, pupil diameter at maximum constriction within pulse on period mean paired difference 0.8%, p = 0.09, mean rate of pupil redilation whilst pulse on mean paired difference 0.16%s^-1^, p = 0.28). These similar initial pupil responses are expected because we deliberately matched contrast between the two stimuli, but differ qualitatively from prior work, which indicate a less sustained pupil response during the stimulus for the melanopsin-directed stimuli, and a faster redilation for the LMS-directed stimuli [13].

All correlations between pupil response parameters and visual field Mean Deviation, visual field Pattern Standard Deviation and mean circumpapillary retinal nerve fibre layer thickness were weak, and none reached statistical significance (Spearman’s rho 0.02 to 0.57).

**Fig 4:**
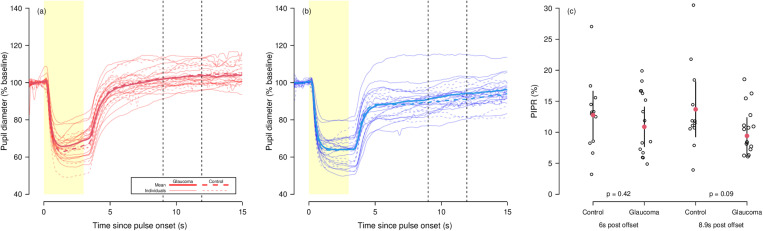
(a) Pupil responses to red and blue stimuli for all participants. Solid lines represent glaucoma participants, dashed lines represent control participants. Bold lines are group means, while fainter lines are individual participants’ mean responses. Red and blue lines represent responses to red and blue stimuli respectively. Vertical dashed lines show the timepoints at which PIPR was compared between groups – 6s and 8.9s after stimulus offset. (b) Post-illumination pupil response (PIPR, red diameter – blue diameter) calculated at 6s and 8.9s post stimulus offset for all participants. Individual participants are shown as open circles, while filled red circles represent group means. Lines represent 95% confidence intervals. P-values shown are for the comparison between groups at each timepoint.

Fig 4a shows the pupil responses to the red and blue stimuli for all participants. Mean PIPR (red-blue) at 6s post stimulus offset was 12.8% (95% confidence interval 8.9–16.6%) for the control group and 10.9% (95% confidence interval 7.7–14.1%) for the glaucoma group (p = 0.42). The timepoint at which the between-group difference in mean PIPR was greatest was 8.9s post stimulus offset (11.9s post stimulus onset). At this timepoint mean PIPR was 13.7% (95% confidence interval 9.3–18.1%) for the control group and 9.4% (95% confidence interval 6.5–12.4%) for the glaucoma group (p = 0.09). Individual and group mean PIPR measurements at both timepoints are shown in Fig 4b.

Diagnostic accuracy for all parameters as measured by area under the ROC curve was poor (Table 4). All parameters except the time taken to reach maximum pupil constriction for the LMS-directed stimulus had 95% confidence interval of the area under the curve overlapping with 0.5, representing performance not significantly better than chance. Time taken to reach maximal pupil constriction for the LMS-directed stimulus had 30% sensitivity at 93% specificity.

**Table 4:** Diagnostic accuracy (area under ROC curve) for detection of glaucoma by all measured parameters. C.I. = confidence interval.

	Area under ROC curve	Lower 95% C.I.	Upper 95% C.I.
Melanopsin-directed stimulus:			
(a)Time to max. constriction	0.60	0.40	0.80
(b)Diameter at max. constriction	0.53	0.33	0.73
(c)Redilation rate	0.60	0.41	0.80
(d)Diameter 6s post-offset	0.51	0.31	0.72
LMS-directed stimulus:			
(a)Time to max. constriction	0.72	0.54	0.90
(b)Diameter at max. constriction	0.61	0.42	0.80
(c)Redilation rate	0.55	0.35	0.76
(d)Diameter 6s post-offset	0.56	0.35	0.78
PIPR:			
(a)Diameter 6s post-offset	0.55	0.33	0.78
(b)Diameter 8.9s post-offset	0.69	0.48	0.89

## Discussion

This study aimed to investigate whether pupillometric measures of melanopsin-driven ipRGC responses may be useful for diagnosing glaucoma. Our silent substitution method allowed us to present pairs of spectral stimuli directed to elicit minimal response from cone photoreceptors while providing sufficient contrast on melanopsin-based ipRGCs to generate a pupil response driven by these cells. The persistent post-illumination pupil constriction with the melanopsin-directed stimuli compared to those to cone photoreceptor-targeted stimuli are consistent with prior data on pupil responses to melanopsin-directed stimuli [13] and, therefore, suggest that our stimuli were successful in differentially stimulating the melanopsin cells.

Differences in pupil responses to melanopsin-directed stimuli between our glaucoma and control groups were minimal and not statistically significant for any of the tested parameters. Similarly, we found no significant differences between the groups in any of the parameters tested for LMS-directed stimuli, nor for 6s or 8.9s PIPR using narrowband red and blue light stimuli. This similarity occurred for the pupillometric measures despite the groups being easily separable based on conventional clinical OCT and visual field tests due to the inclusion criteria. Our data, therefore, suggest that pupillometric measurements of melanopsin-driven ipRGC function are not likely to be clinically useful as diagnostic biomarkers of glaucoma.

To our knowledge, this is the first study to investigate pupil responses to melanopsin-directed silent substitution stimuli in glaucoma. Previous studies have measured the PIPR using narrowband red/blue stimuli, finding statistically significant between-group differences [4–8]. In our study, this difference was not statistically significant (p = 0.42), but a larger difference in PIPR was found at 8.9s post stimulus offset (Fig 4). This difference did not quite reach statistical significance (p = 0.09), though we note that our sample size for this comparison is smaller than some previous studies and a comparable sample size to previous studies may have rendered an effect of this size statistically significant. There are other differences between our study and previous studies of the PIPR to narrowband red/blue stimuli that may also have contributed to the different result. We used an integrating sphere rather than a Maxwellian-view or similar system as in most previous studies [4–8]. These previous studies also stimulated through one pupil, which was typically pharmacologically-dilated, whilst measuring the consensual pupil responses of the unstimulated fellow eye [4–8]. We did not use any pharmacological dilation as we measured the direct pupil responses of the stimulated eye whilst the fellow eye was occluded. Whilst this may have contributed to some differences in the form of the pupil response, perhaps explaining the different time-point of maximum between-group PIPR difference, we do not think this explains any reduction in between-group differences. We note that we are not the first to use a type of integrating sphere along with a head-mounted pupillometer, as our method is broadly similar to that of Najjar et al. who used a similar pupillometer in conjunction with a Ganzfeld bowl system to measure pupil responses to narrowband stimuli [9].

In our data the narrowband PIPR stimuli came closer to achieving both a statistically-significant between-group mean difference and a diagnostic accuracy significantly better than chance, at least for the 8.9s post offset timepoint, than did any of the melanopsin-directed stimulus parameters. One possible explanation is that responses to the narrowband stimuli also include a significant rod component (as they are pulsed from the dark), and overall have larger, but non-isolating, contrast on the melanopsin system. As our silent-substitution method used stimulus contrasts consistent with prior studies, reaching the near-limit of contrast for multi-primary systems, a further optimisation of silent-substitution stimulus parameters may not be feasible [13,25].

The small sample size is a limitation of our study in detecting between-group differences. However, the purpose of our study was not simply to detect a mean difference between groups, but rather to test whether this technique has potential for use as a diagnostic test for glaucoma. Between-group differences in mean parameters are not sufficient to demonstrate clinical utility as a diagnostic test. Therefore, though a larger sample may have rendered small between-group differences statistically significant, adding further participants to the sample would be unlikely to change the conclusion that these measures are not suitable for use as a diagnostic test. Diagnostic performance in this study was close to chance, meaning that even a larger sample using these methods would be unlikely to yield an acceptable performance. For example, to achieve even a modest 70% sensitivity at 90% specificity requires the means of two normally distributed groups to be separated by 1.8 standard deviations (Cohen’s d = 1.8). Our study had more than 99% power to detect differences of this size with alpha = 0.05 at the achieved sample sizes, and therefore even greater power to detect the greater between-group separation required to attain the higher sensitivity and specificity needed for a clinical glaucoma test.

It is possible that between-group differences would have been larger had we recruited participants with more advanced glaucoma. This has been the case in previous studies showing either a greater difference in more advanced disease [7], not showing a significant difference in early disease but doing so in advanced disease [4], or only including advanced cases in the glaucoma group [5]. The visual field mean deviation in our glaucoma group was mean -5.72dB (standard deviation 2.67dB) which represents moderate loss, but is still less than half of the mean in Kankipati et al. [5] or the “Late” group in Adhikari et al. [7] A further histological study found melanopsin-based RGC loss in severe glaucoma eyes, but not in early glaucoma eyes post-mortem [3]. The participants in our glaucoma group were, through the inclusion criteria, already readily detected by conventional visual field and OCT diagnostic tests (both would have an area under the ROC curve of 1.0). Therefore, any new diagnostic test that only picks up more advanced cases than these would be of limited clinical use.

It is worth considering whether changes to the stimulus or protocol may be beneficial in eliciting a diagnostically-useful effect. Our pilot data suggested that our PIPR results did not differ with or without 30 minutes of dark adaptation, and we used 4.5 minutes of dark adaptation in our protocol. Some previous studies have used longer periods (e.g., 10 minutes) of dark adaptation in eliciting a strong PIPR [7]. The aim of our study was to test potential as a clinical diagnostic test, and the requirement for long periods of dark adaptation limits the clinical applicability of a test. A further modifiable parameter is the pulse duration. We chose a 3s stimulus pulse as used successfully by McAdams et al. [13], but Park et al. [23] found that a 1s pulse elicited a larger PIPR than a 3.16s pulse in healthy subjects. It is therefore possible that a shorter pulse, with or without additional dark adaptation may have elicited a greater between-group difference. For the red/blue narrowband stimuli, the protocol used means that it is possible that some sustained pupil constriction from the blue stimulus persisted into trials with the red stimulus. This may have affected the size of the red-blue PIPR found in some participants, however this counterbalanced design has been previously successful [13]. We re-iterate that, even in these groups of participants easily distinguished by current imperfect clinical tests, none of our metrics performed close to a clinically-useful level in terms of sensitivity/specificity or ROC area (Table 4). Therefore, whilst some changes to the stimulus or protocol may improve the performance, and may yield further statistically significant differences between group means, improvements would have to be greater than we would expect in order to achieve the high levels of sensitivity and specificity required for clinical glaucoma diagnosis.

A further limitation of our method is that we did not adjust the stimuli to account for individual differences in ocular media transmission. Particularly, 8 of the glaucoma participants and 2 of the control participants had artificial intraocular lenses following cataract surgery. We considered that variations in ocular transmission, which are also affected by age-related changes in the cornea and vitreous, are a contributory factor to between-individual variation that a clinical test must overcome. Nevertheless, we did perform a post-hoc comparison of the responses to the melanopsin-directed stimulus between the glaucoma participants who had not had cataract surgery to those of the control group. Although this comparison is less well-controlled and lower powered than the main analysis, the results were similar to those in Table 3, with no statistically significant differences in these parameters. A clinical test could potentially offer different settings for participants with/without intraocular lenses and by age, and this may be worthy of further study if spectral settings can be found that substantially reduce between-individual variability.

In conclusion, we have measured pupil responses to melanopsin-directed silent substitution stimuli in age-similar participants with and without conventionally-diagnosed glaucoma. The pupil responses to melanopsin-directed stimuli were similar between groups and thus are unlikely to be suitable as a basis for diagnostic tests for glaucoma. Further studies may evaluate the efficacy of similar approaches in other conditions, such as outer retinal disease, where results may differ.

## Supporting information

S1 Fig Spectral response of the stimulation system. (left) Spectral response of the stimulation system measured at the corneal plane for each of the independent primaries, and (right) the gamut of the device plotted on the CIE 1931 chromaticity horseshoe.
(DOCX)

S2 Fig Boxplots of pupil diameters for each group/stimulus during the baseline period of 1s prior to stimulus onset. Pupil diameters during the baseline period within 1s prior to stimulus onset for all stimuli. Boxplots marked G represent the glaucoma group, while those marked C represent the control group. Bold black lines show group medians, boxes show interquartile range, whiskers show full range excluding any outliers. Outliers (defined as points > 1.5x the interquartile range away from nearest quartile) are shown as individual points. Mel = melanopsin-directed stimulus, LMS = LMS-directed stimulus, Red = narrowband red stimulus, Blue = narrowband blue stimulus. It should be noted that during the background period there was no stimulus (i.e., dark) for the red/blue stimuli, whereas for the Melanopsin- and LMS- directed stimuli the background stimulus was shown, hence the smaller pupil diameters for those stimuli.
(DOCX)

S1 File controlBlueStimulus. (CSV)

S2 File controlRedStimulus. (CSV)

S3 File controlLMSStimulus. (CSV)

S4 File controlMelanopsinStimulus. (CSV)

S5 File glaucomaBlueStimulus. (CSV)

S6 File glaucomaRedStimulus. (CSV)

S7 File glaucomaLMSStimulus. (CSV)

S8 File glaucomaMelanopsinStimulus: These files (all in the same format) contain the pupil measurements for each participant and each stimulus as indicated in the file name. Pupil measurements are processed as described in the manuscript (fitted with 3-D model, blinks removed, smoothed) and normalised to baseline pupil measurement (mean pupil diameter within 1s prior to stimulus onset). Pupil measurements are therefore given in %, where 100% = baseline diameter. Columns showing NA indicate missing data (i.e., that participant did not contribute data for that stimulus). The column labelled “timeRelativeToPulseOnset” gives time relative to pulse onset (where negative time means prior to onset) in seconds. Subsequent columns each represent a participant named according to the column heading.
(CSV)

S9 File Mean Stimulus Radiances. This file contains the spectral radiances for all stimuli. Data provided are the means of each stimulus measurement taken before and after each data collection session (5 before, 5 after each session). Columns as follows: ‘wavelength’ – wavelength in nm. MelPulse – radiance of pulse for Melanopsin-directed stimulus pair in Wm^-2^sr^-1^nm^-1^. MelBg – radiance of background for Melanopsin-directed stimulus pair in Wm^-2^sr^-1^nm^-1^. LMSPulse – radiance of pulse for LMS-directed stimulus pair in Wm^-2^sr^-1^nm^-1^. LMSBg – radiance of background for LMS-directed stimulus pair in Wm^-2^sr^-1^nm^-1^. red – radiance of pulse for narrowband red stimulus in Wm^-2^sr^-1^nm^-1^. blue – radiance of pulse for narrowband blue stimulus in Wm^-2^sr^-1^nm^-1^.
(CSV)
