# Supplementary figures and images for: Pupil responses to melanopsin-isolating stimuli as a potential diagnostic biomarker for glaucoma
Source: PLoS One. 2025 May 23;20(5):e0324373. doi: 10.1371/journal.pone.0324373 (PMC12101769; doi:10.1371/journal.pone.0324373)

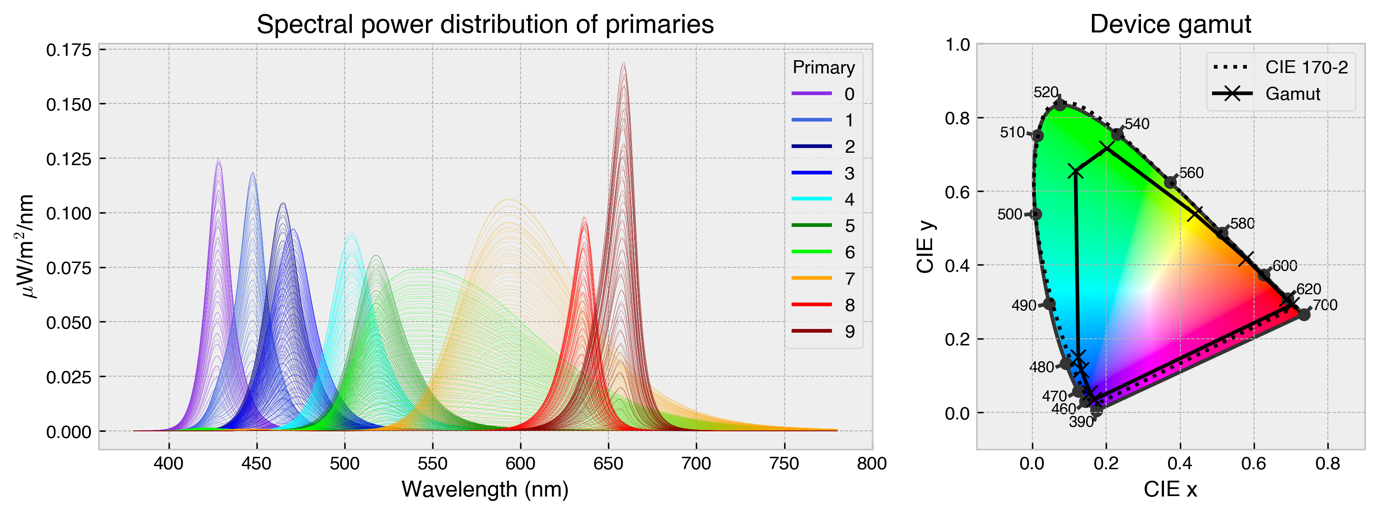

Supplement: S1 Fig — (left) Spectral response of the stimulation system measured at the corneal plane for each of the independent primaries, and (right) the gamut of the device plotted on the CIE 1931 chromaticity horseshoe. (DOCX) [file pone.0324373.s001.docx]
