# Supplementary material for: Pupil responses to melanopsin-isolating stimuli as a potential diagnostic biomarker for glaucoma
Source: PLoS One. 2025 May 23;20(5):e0324373. doi: 10.1371/journal.pone.0324373 (PMC12101769; doi:10.1371/journal.pone.0324373)
Supplement: S2 Fig — Pupil diameters during the baseline period within 1s prior to stimulus onset for all stimuli. Boxplots marked G represent the glaucoma group, while those marked C represent the control group. Bold black lines show group medians, boxes show interquartile range, whiskers show full range excluding any outliers. Outliers (defined as points > 1.5x the interquartile range away from nearest quartile) are shown as individual points. Mel = melanopsin-directed stimulus, LMS = LMS-directed stimulus, Red = narrowband red stimulus, Blue = narrowband blue stimulus. It should be noted that during the background period there was no stimulus (i.e., dark) for the red/blue stimuli, whereas for the Melanopsin- and LMS- directed stimuli the background stimulus was shown, hence the smaller pupil diameters for those stimuli. (DOCX) [file pone.0324373.s002.docx]

Supplementary Figure 2:

Pupil diameters during the baseline period within 1s prior to stimulus onset for all stimuli. Boxplots marked G represent the glaucoma group, while those marked C represent the control group. Bold black lines show group medians, boxes show interquartile range, whiskers show full range excluding any outliers. Outliers (defined as points >1.5x the interquartile range away nearest quartile) are shown as individual points. Mel=melanopsin-directed stimulus, LMS=LMS-directed stimulus, Red=narrowband red stimulus, Blue=narrowband blue stimulus.

It should be noted that during the background period there was no stimulus (i.e. dark) for the red/blue stimuli, whereas for the Melanopsin- and LMS- directed stimuli the background stimulus was shown, hence the smaller pupil diameters for those stimuli.
